# An exhaustive examination of the research progress in identifying potential JAK inhibitors from natural products: a comprehensive overview

**DOI:** 10.1186/s13020-025-01176-0

**Published:** 2025-08-21

**Authors:** Wendong Yang, Jiabin Lu, Peihua Luo, Zhifei Xu, Hao Yan, Bo Yang, Qiaojun He, Jialin Zhou, Xiaochun Yang

**Affiliations:** 1 Center for Drug Safety Evaluation and Research, College of Pharmaceutical Sciences, Zhejiang University, Hangzhou, 310058 China; 2 Nanhu Brain-Computer Interface Institute, Hangzhou, 311100 China; 3 Innovation Institute for Artificial Intelligence in Medicine of Zhejiang University, College of Pharmaceutical Sciences, Zhejiang University, Hangzhou, 310058 China; 4 Hangzhou Institute of Innovative Medicine, College of Pharmaceutical Sciences, Zhejiang University, Hangzhou, 310058 China; 5 School of Medicine, Hangzhou City University, Hangzhou, 310015 China; 6 Tiansure Pharmaceutical Co., Ltd, Hangzhou, 311051 China

**Keywords:** JAK-STAT pathway, JAK inhibitors, Natural products, Inflammatory diseases, Autoimmune diseases, Malignant tumors

## Abstract

The JAK-STAT signaling pathway serves as a central regulator of diverse cellular processes encompassing proliferation, apoptosis, inflammation, and differentiation. Specifically, extracellular ligands such as interleukins, and colony-stimulating factors induce JAKs phosphorylation, subsequently triggering dimerization and nuclear translocation of STATs protein. In this way, the JAK-STAT pathway modulates target gene expression. Dysregulation of the JAK-STAT pathways has been implicated in the pathogenesis of multiple diseases, including inflammatory diseases, autoimmune diseases, malignant tumors. Therefore, JAK inhibitors have been considered promising therapeutic candidates with substantial clinical potential. While previous reviews have primarily focused on natural products targeting JAK-STAT signaling pathways for the specific disease application, this paper comprehensively collected 88 natural products demonstrating JAKs inhibitory activity across multiple pathological conditions. We mainly referenced nearly 20 years of literature from 2005 to 2025, comprising 294 different types of publications including review articles and research papers. Through systematic analysis of the compounds, we further classified these phytochemicals according to their structural characteristics (flavonoids, alkaloids, terpenoids) and molecular targets within the signaling cascades. This study provides novel insights into the pathophysiological relationships between diseases and JAK kinases, while offering valuable guidance for developing next-generation JAK inhibitors with improved therapeutic profiles.

**Graphical abstract:**

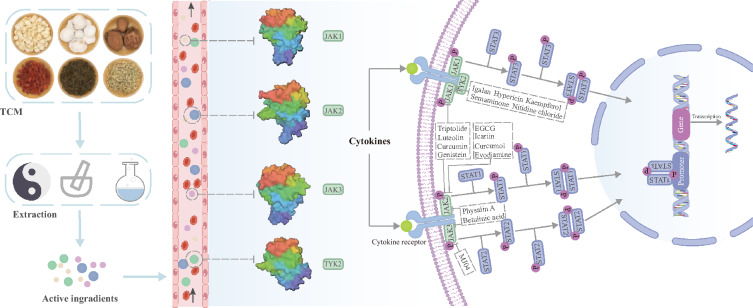

**Supplementary Information:**

The online version contains supplementary material available at 10.1186/s13020-025-01176-0.

## Background

**Fig. 1:**
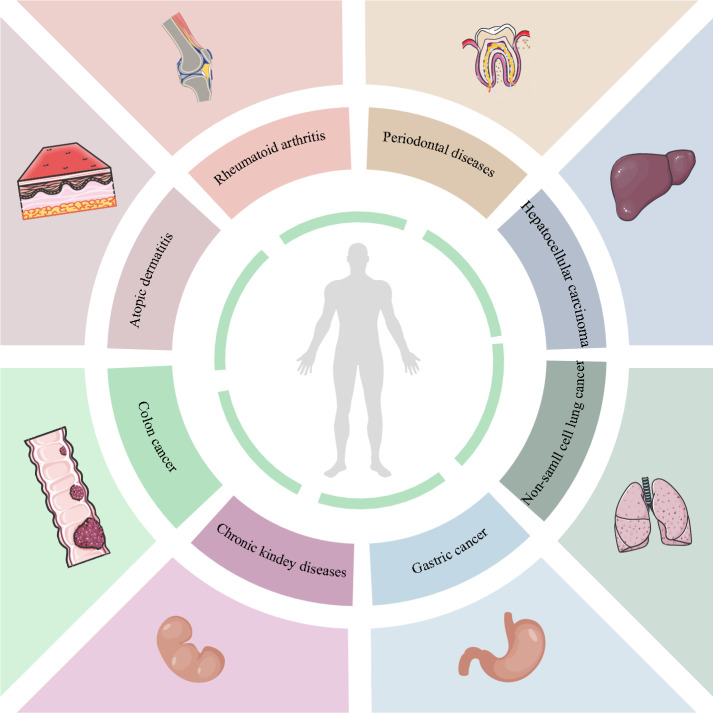
JAK-STAT pathway-associated disorders and their target organ systems. Eight clinically significant disease entities demonstrate established associations with JAK-STAT signaling abnormalities: atopic dermatitis, rheumatoid arthritis, periodontal diseases, hepatocellular carcinoma, non-small cell lung cancer, gastric cancer, chronic kidney disease and colon cancer

The JAK (Janus Kinase)-STAT (Signal Transducer and Activator of Transduction) pathway functions as a central signaling nexus that coordinates rapid transduction of extracellular signals from membrane receptors to nuclear effectors. The tyrosine kinase family comprises four structurally homologous members, namely JAK1, JAK2, JAK3, TYK2 [1]. Mechanistic studies have established that dysregulated JAK activity is pathologically implicated in autoimmune disorders and oncogenesis [2]. Notably, JAK-STAT hyperactivation drives the inflammatory cascade in atopic dermatitis (AD), positioning JAK inhibitors (e.g., tofacitinib, baricitinib) as first-line therapeutic agents for moderate-to-severe AD [3, 4]. Similarly, pro-inflammatory and anti-inflammatory cytokines central to rheumatoid arthritis (RA) can be produced additionally through the JAK-STAT pathway, explaining the clinical efficacy of JAK inhibition in RA management [5]. An overview of diseases correlated with the JAK-STAT pathway and organs where the diseases occur is presented below (Fig. 1).

Currently approved JAK inhibitors including tofacitinib and baricitinib demonstrate significant clinical efficacy, particularly in managing cutaneous inflammatory disorders. Nevertheless, three clinically significant adverse events—opportunistic infections, acquired drug resistance, and thromboembolic complications—remain critical concerns [6–8]. Therefore, these limitations collectively underscore the critical imperative to engineer next-generation JAK inhibitors with enhanced therapeutic safety.

Traditional Chinese medicine (TCM) represents a unique therapeutic paradigm characterized by unparalleled chemical diversity and pharmacological diversity. Accumulating empirical validation through long-term clinical practice has led to growing global recognition of its therapeutic value. Notably, studies have systematically confirmed that TCM-derived agents possess distinct antitumor and immunomodulatory properties [9–11]. Based on this pharmacological foundation, our investigation prioritizes traditional herbal compounds as a strategic resource for JAK inhibitor discovery, aiming to identify novel small-molecule candidates from TCM’s chemically diverse repository.

## Traditional utilization of TCM and natural products from TCM

With documented use spanning millennia, TCM stands as one of humanity’s earliest systematized therapeutic approaches. The integration of TCM’s empirical knowledge with modern target discovery constitutes an efficient discovery paradigm, as evidenced by multiple pharmacological validations. Representative examples include: *Tripterygium wilfordii* Hook F, historically employed in managing immune-related rheumatic diseases, has demonstrated extended therapeutic value in non-small cell lung cancer (NSCLC) treatment [12]; *Crocus sativus* L., historically utilized in Asian anticancer regimens, has yielded the bioactive constituent crocin demonstrating marked efficacy against gastric malignancies [13, 14]; *Psoralea corylifolia* L., a canonical osteoprotective agent, contains isobavachalcone with retained therapeutic efficacy in RA [15, 16]; *Hypericum perforatum* L., traditionally applied for burns, sunburns, and gastric irritation, has been confirmed hypericin-mediated anti-inflammatory property [17, 18]; *Reseda odorata* L., with traditional applications against oxidative stress and acute inflammation, provides luteolin showing clinical potential in ulcerative colitis and AD [19, 20]. Mechanistic studies consistently identify JAK inhibition as the unifying pharmacological mechanism underlying these therapeutic outcomes. These cases collectively demonstrate the promise of identifying JAK inhibitors from TCM for broader disease treatment.

**Fig. 2:**
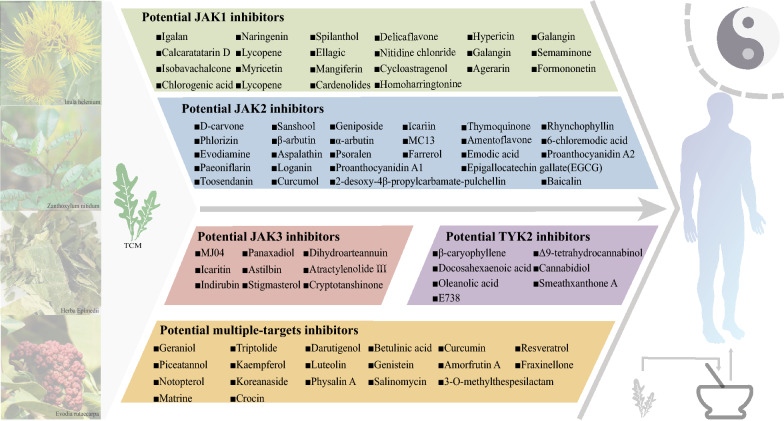
Mechanistic classifications of JAK inhibitors derived from TCM. The left panel depicts four flavors of TCM. The center panel depicts potential JAK inhibitors. The right panel depicts the concept of the TCM philosophy. Natural products with known chemical structures can be classified as potential JAK1 inhibitors, potential JAK2 inhibitors, potential JAK3 inhibitors, potential TYK2 inhibitors and potential multiple-targets inhibitors. JAK, Janus kinase; TYK2, Tyrosine kinase 2; TCM, Traditional Chinese medicine

Current JAK inhibitor discoveries from natural products show uneven distribution across subtypes. Our review found more JAK1 and JAK2 inhibitors than JAK3 and TYK2 inhibitors, possibly due to their different discovery periods. We have categorized these natural products by targets (Fig. 2).

## Potential JAK1 inhibitors from natural products

**Fig. 3:**
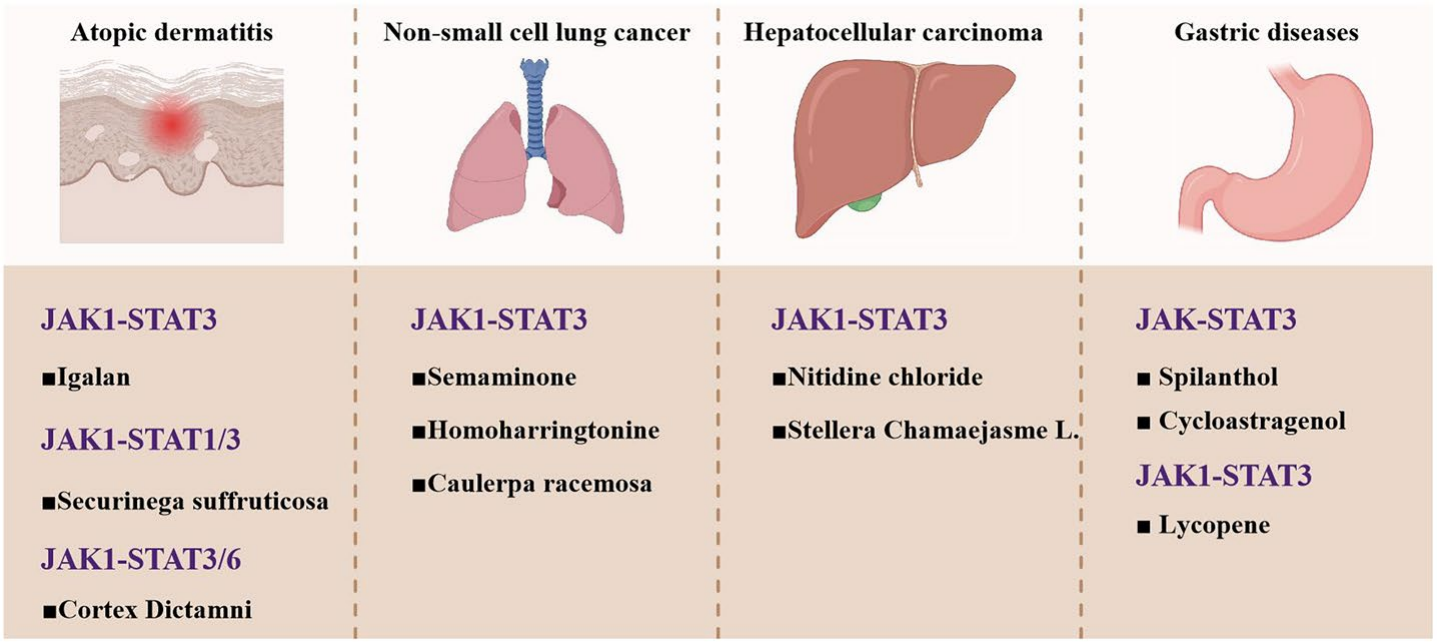
Four JAK1-associated diseases and potential therapeutic JAK1 inhibitors with signaling pathways. Four clinically relevant conditions demonstrating JAK1-STAT3 pathway involvement: atopic dermatitis, non-small cell lung cancer, hepatocellular carcinoma and gastric disorders. The JAK1-STAT3 signaling pathway is shown as the mechanistic component shared across all listed conditions. JAK, Janus kinase; STAT, signal transducer and activator of transcription

This section profiles 19 structurally characterized natural products exhibiting JAK1 inhibitory potential. These natural products encompass four principal chemotypes: terpenes, polyphenols, phenylpropanoids and alkaloids, with polyphenols representing the predominant subclass and demonstrating proportionally broader therapeutic potential. Therapeutic indications span AD, RA, gastric cancer, NSCLC and others (Fig. 3). A visual demonstration of the chemical names, CAS numbers, chemical structures, species, classifications, IC_50_, signaling pathways or targets, pharmacological effects or applications of the natural compounds is provided (Table 1).

**Table 1:** Potential JAK1 inhibitors with known structures from natural products

Candidates CAS number	Chemical structures	Species	Classifications	IC_50_	Signaling pathways/targets	Pharmacological effects/applications
Igalan 97456–58-1		*Inula helenium* L	Sesquiterpenes	< 5 μM	JAK1-STAT3; Nrf2	Atopic dermatitis
Calcaratarin D **–**		*Alpinia calcarata* (Haw.) Roscoe	Diterpenes	< 100 μM	Nrf2-HO-1; FoxO1-IRF4; JAK1-STAT6	Asthma
Spilanthol 25,394–57-4		*Acmella oleracea* (L.) R.K.Jansen	Diterpenes	–	JAK1/2-STAT3	Gastric cancer
Cycloastragenol 78,574–94-4		*Astragalus membranaceus* (Fisch.) Bunge	Triterpene saponins	< 50 μM	JAK1/Src-STAT3	Gastric cancer
Ouabain 11,018–89-6		*Strophanthuk kombe* Oliv	Steroids	< 100 nM	JAK1-STAT1/3	Gastroenteritis coronavirus
Lycopene 502–65-8		*Solanum lycopersicum* L	Carotenoids	< 1 μM	JAK1-STAT3; Wnt-β-catenin	Gastric diseases
Ellagic Acid 476–66-4		*Rubus cochinchinensis* Tratt.; *Carya cathayensis* Sarg	Polyphenols	–	JAK1/JAK2-STAT1-NOX4	Renal ischemic-reperfusion injury
Naringenin 67,604–48-2		*Anacardium occidentale* L	Isoflavonoids	–	TP53; CASP3; PI3K-AKT; MAPK-ERK; NF-κB; JAK1-STAT3	Nonalcoholic fatty liver disease
Isobavachalcone 20,784–50-3		*Cullen corylifolium* (L.) Medik	Isoflavonoids	< 20 μM	PI3K-AKT; JAK1-STAT3	RA
Delicaflavone 343,569–15-3		*Selaginella doederleinii* Hieron	Isoflavonoids	< 1.25 μg/mL	JAK1-STAT6	Tumor immune responses
Galangin 548–83-4		*Alpinia calcarata* (Haw.) Roscoe	Isoflavonoids	< 25 μM	IRAK-1; MAPK; NF-κB; JAK1-STAT	Inflammatory responses
Myricetin 68,708–52-1		*Myrica rubra* (Lour.) Siebold & Zucc	Isoflavonoids	< 20 μM	JAK1-STAT3	Inflammatory responses; Cardiovascular pathologies; Cancer
Mangiferin 4773–96-0		*Mangifera indica* L	Xanthones	–	NF-κB; JAK1-STAT1/3	Periodontitis
Formononetin 485–72-3		*Astragalus membranaceus* (Fisch.) Bunge	Isoflavones	< 100 μM	JAK1/2-STAT3/5; ERK	Cervical; Lung cancers; Colorectal cancers, Breast cancers
Hypericin 548–04-9		*Hypericum perforatum* L	Anthraquinones	–	JAK1-STAT	Inflammatory responses
Chlorogenic acid 1,049,703–62-9		*Lonicera japonica* Thunb	Phenylpropanoids	> 25 μM	JAK1-STAT3; NF-κB; Nrf2-HO-1	RA
Semaminone -		*Zanthoxylum nitidum* (Roxb.) DC	Lignans	2.5–5 μM	JAK1-STAT3; PI3K-AKT	NSCLC
Nitidine chloride 13,063–04-2		*Zanthoxylum nitidum* (Roxb.) DC	Alkaloids	–	JAK1-STAT3	HCC
Homoharringtonine 26,833–87-4		*Cephalotaxus harringtonia* var. *fastigiata* (Carrière) Rehder	Alkaloids	< 1 μM	JAK1-STAT3	NSCLC

### Terpenes

#### Sesquiterpenes

*Inula helenium* (L.), a medicinal plant with established anti-inflammatory properties, yields the sesquiterpene lactone Igalan, which demonstrates therapeutic potential for AD. Mechanistic studies indicated that Igalan ameliorated epidermal barrier dysfunction through dose-dependent JAK1 inhibition. And Igalan concomitantly downregulating IL-4Rα and IL-13Rα expression, thereby attenuating JAK1-STAT3 signaling [21]. Further investigation should focus on Igalan’s molecular targets and translational applications in AD management.

#### Diterpenes

Calcaratarin D (CalD), a bioactive diterpenoid isolated from *Alpinia calcarata* (Haw.) Roscoe rhizomes, inhibited IL-4 and IL-13-induced JAK1-STAT6 activation, thereby modulating M2-like phenotype polarization and suppressing Th2 cytokine secretion [22]. While JAK1 is not the sole target of CalD in regulating alveolar macrophage function, the potential of CalD in asthma merits further investigation.

#### Triterpene saponins and steroids

Phytochemicals exhibit dual therapeutic roles in oncology, functioning both as nanotherapeutics and combination partners. Cycloastragenol (CAG), a triterpenoid aglycone derived from *Sigesbeckia orientalis* L. roots, inhibited constitutive STAT3 activation through JAK1 and Src kinase suppression. Synergistic cytotoxicity emerged when CAG combined with paclitaxel, demonstrating enhanced growth inhibition in gastric adenocarcinoma cells compared to monotherapy [23]. This synergy highlights the emerging paradigm of phytochemical-based combination regimens in oncology therapy.

Ouabain, a cardenolides characterized by a steroidal scaffold fused with a lactone ring, exhibited antiviral specificity through selective JAK1 downregulation via a Na–K-ATPase-independent proteolysis [24]. Notably, as transmissible gastroenteritis coronavirus infection demonstrated negligible modulation of JAK2, JAK3 and TYK2, whether Ouabain interacts with other JAK members remains uncertain.

#### Carotenoids

Lycopene, a lipophilic carotenoid pigment predominantly isolated from *Solanum lycopersicum* L., demonstrated potent antioxidant capacity and broad-spectrum anticancer activity across 12 solid tumor types [25, 26]. Mechanistically, Lycopene suppressed *Helicobacter* *pylori*-induced JAK1-STAT3 hyperactivation in gastric epithelial cells, thereby preventing *Helicobacter* *pylori*–associated carcinogenesis [26].

### Polyphenols

Plant polyphenols have attracted significant research interest for their notable anti-inflammatory properties. Studies have demonstrated that a bound polyphenol extract derived from insoluble dietary fiber of *Rubus cochinchinensis* Tratt effectively alleviated cellular inflammation by inhibiting the LPS-induced JAK1-STAT3 pathway in RAW264.7 macrophages [27]. Ellagic acid, a polyphenol dilactone compound, demonstrated therapeutic potential by inhibiting the phosphorylation of JAK1, JAK2, and STAT1, while concurrently suppressing the level of NOX4, thus ameliorating renal ischemic-reperfusion injury [28].

#### Isoflavonoids

Naringenin, a dihydroflavonoid compound, exhibited multifaceted therapeutic effects, including: anti-inflammatory, DNA-protective, hypolipidemic, antioxidant and PPARγ agonist activity [29–31]. Preclinical studies have provided mechanistic evidence that naringenin held therapeutic promise for nonalcoholic fatty liver disease through selective inhibition of JAK1 [31].

Isobavachalcone (IBC), a natural chalcone compound, is pharmacologically active in multiple traditional Chinese medicinal plants, including *Cullen corylifolium (L.)* Medik, *Morus alba* L., and others. Network pharmacology analysis revealed that IBC’s therapeutic effects on RA may involve dual modulation of the PI3K-AKT and JAK1-STAT3 signaling pathways, which collectively regulated inflammatory cell proliferation and apoptosis resistance. This hypothesis was subsequently validated by cell-based experiments [15].

*Selaginella doederleinii* Hieron, an ethnomedicinal plant indigenous to Southern China and Southeast Asia, demonstrated multifaceted pharmacological properties, including anti-proliferation, anti-oxidation, and anti-Alzheimer and anti-tumorigenesis [32]. Delicaflavone, a biflavonoid compound purified from *S. doederleinii*, selectively inhibited the JAK1-STAT6 signaling pathway in M2-polarized tumor-associated macrophages and myeloid-derived suppressor cells, effectively reprogramming immunosuppressive tumor microenvironments to restore anti-tumor immunity [32].

*Alpinia calcarata* (Haw.) Roscoe, a medicinal rhizome in the Zingiberoside family, has been traditionally used for inflammatory disorders. Galangin, the principal bioactive flavonoid isolated from this plant, selectively inhibited JAK1 phosphorylation in LPS-stimulated RAW 264.7 cells, validating its anti-inflammatory efficacy [33].

Myricetin is a ubiquitous dietary flavonoid abundant in *Myrica rubra* (Lour.) Siebold & Zucc, fruits, vegetables, berries and red wine. Prior research has demonstrated that myricetin bound with high specificity to both JAK1 and STAT3, inhibiting EGF-induced malignant transformation in the JB6 P + mouse epidermal cell model. Moreover, myricetin exhibited a higher affinity for JAK1 than STAT3 [34, 35].

#### Xanthones

Mangiferin, a xanthonoid polyphenol with anti-inflammatory properties, had limited reported evidence regarding its JAK1-targeting activity Periodontitis, the most prevalent oral infection in humans and primary cause of adult tooth loss, has been investigated in murine models. Mangiferin treatment significantly reduced the phosphorylation levels of JAK1, STAT1 and STAT3 in gingival epithelia of periodontitis mice. Oral administration of Mangiferin also attenuated alveolar bone loss, demonstrating therapeutic potential [36].

#### Isoflavones

Formononetin (FT), a natural isoflavone isolated from *Astragalus membranaceus* (Fisch.) Bunge, demonstrated broad-spectrum antitumor activity against multiple malignancies including multiple myeloma, cervical, lung, colorectal, and breast cancers [37–39]. Preclinical study in multiple myeloma models revealed that FT suppressed the STAT3/5-DNA binding capacity while simultaneously inhibiting the activation of upstream kinases JAK1, JAK2 and Src [37]. In another report, FT-mediated STAT3 inhibition via the JAK1-STAT3 axis reduced the PD-L1 suggesting immune-modulatory effects. Collectively, these findings establish the JAK-STAT pathway as FT’s primary antitumor mechanism [39].

#### Anthralones

Hypericin, an anthraquinone derivative predominantly found in *Hypericum perforatum* L., It demonstrated biological activities including anti-tumor, anti-viral, neuroprotective effects in depression, Alzheimer’s disease and autism spectrum disorder [40, 41]. A computational analysis identified JAK1 as the principal molecular target mediating its anti-inflammatory property [17].

### Phenylpropanoids

#### Phenylpropanoids

Chlorogenic acid (CGA), the principal bioactive constituent of the traditional Chinese herb *Lonicera japonica* Thunb, is clinically employed in RA management [42]. This phenolic compound exhibited dual anti-inflammatory and anti-oxidant properties [43, 44]. Prior research demonstrated that CGA markedly inhibited IL-1β/IL-6-mediated proliferation of RSC-364 cells through pro-apoptotic effects and downregulation of gp130, JAK1 and STAT3 [42].

#### Lignans

Semaminone, a tetrahydrofuran lignan, was isolated from *Zanthoxylum nitidum* (Roxb.) DC. Semaminone downregulated the activation of JAK1-STAT3 pathway, inhibiting proliferation of osimertinib-resistant EGFR-mutant NSCLC cells [45]. The combination of Semaminone and osimertinib demonstrated a synergistic growth inhibition in hepatocellular carcinoma (HCC) 827-osi resistant cells, suggesting a potential salvage therapy for EGFR mutation-mediated osimertinib resistance in NSCLC.

### Alkaloids

HCC ranks as the sixth most prevalent malignancy and second leading cause of cancer-related mortality worldwide [46]. In HCC xenograft mouse models, treatment with Nitidine chloride (NC) achieved 52% reduction in tumor volume and 41% decrease in tumor weight. Mechanistic studies revealed that NC significantly inhibited JAK1 and STAT3 phosphorylation without significantly affecting their total protein levels [47].

Homoharringtonine (HHT), is a bioactive cephalotaxine ester derived from *Cephalotaxus harringtonia* var. fastigiata (Carrière) Rehder, with demonstrated antitumor activity previous studies have shown that HHT induced mitochondrial-mediated apoptosis through caspase cascade activation and suppressed the JAK-STAT3 pathway in NSCLC cells [48].

### Total extracts

Numerous plant extracts demonstrate JAK1-targeting activity. Our systematic review catalogues 17 crude extracts and their respective species, signaling pathways or targets classification of active ingredients, potential applications and references (Table 2). Notable species include *Solanum* L., *Lonicera* L. and *Stellera* Linn. The selected species represent distinct botanical families, demonstrating substantial phylogenetic diversity among JAK1-targeting plants. Notably, the fungal metabolite ( +)-terrein has been utilized in the treatment of periodontal diseases, emerging as a novel candidate for JAK1 modulation.

**Table 2:** Total extracts target JAK1

Species	Signaling pathways/targets	Classification of active ingredients	Potential applications	Refs.
( +) -Terrein	JAK1-STAT3	Fungal metabolite	Periodontal diseases	[209]
*Annona squamosa* L. (Fruit)	JAK1-STAT3	–	Testicular injury	[210]
*Caulerpa racemosa*	JAK1-STAT3; EGFR	–	NSCLC	[211]
*Dictamnus dasycarpus* Turcz*.* (dry root bark)	PI3K-AKT; JAK1-STAT3/6	Flavonoids	Atopic dermatitis	[212]
*Dendrobium huoshanense* Z.Z.Tang & S.J.Cheng (stem)	NF-κB; MAPK; PI3K-AKT; JAK1-STAT3	Polysaccharides	RA	[213]
*Lycium chinense* Mill*.* (seed oil)	JAK1-STAT1; NF-κB	Essentialoil	Inflammation in testis	[214]
*Hosta plantaginea* (Lam.) Asch*.* (flowers)	NF-κB; MAPK; JAK1-STAT3	Flavonoids	Chronic prostatitis	[215]
*Juniperus rigida* Siebold & Zucc*.* (dried leaves and twigs)	JAK1-STAT1	Polyphenols	Inflammatory responses	[216]
*Leonurus japonicus* Houtt	JAK1-STAT1	–	Intracerebral hemorrhage	[217]
*Lonicera japonica* Thunb*.* (flower buds)	MAPKs; PI3K-AKT; JAK1-STAT1/3	–	Inflammatory responses	[218]
*Marsdenia tenacissima* (Roxb.) Moon (dry caulis)	JAK1-STAT3; HIF-1α; P53	–	Hepatocellular Carcinoma	[219]
*Phoenix dactylifera* L.(seed)	JAK1-STAT3	Polyphenols	RA	[220]
*Securinega suffruticosa* (Pall.) Rehder (leaves)	JAK1-STAT1/3	–	Atopic dermatitis	[221]
*Solanum nigrum* L*.* (Unripe fruit)	JAK1-STAT3; MDR1	Glycosides	Adriamycin resistance	[222]
*Stellera Chamaejasme* L	MiR-134-5p; JAK1-STAT3	–	Hepatocellular Carcinoma	[223]
*Tinospora sinensis* (Lour.) Merr	JAK1-STAT; PI3K-AKT	–	Alzheimer's Disease	[224]

Flavonoids exhibit remarkable performance in structure–activity relationships, likely due to their relatively planar or rigid hydrophobic core facilitating binding with the JAK1 active pocket, thus demonstrating significant potential in developing JAK1 inhibitors. Particularly noteworthy is the activity of flavonoids featuring a 5,7-dihydroxy A-ring and appropriate hydroxyl substitutions on the B-ring. For instance, compounds such as Galangin, Myricetin, and Delicaflavone display potent inhibitory activity. Bulky hydrophobic groups may enhance binding to JAK1 through van der Waals forces; for example, the cardenolide structure of Ouabain and the linear polyene structure of Lycopene confer higher potency to these compounds, though potentially reducing binding specificity. The number of carbonyl and hydroxyl groups within a compound also influences its inhibitory activity and selectivity against JAK1.

## Potential JAK2 inhibitors from natural products

**Fig. 4:**
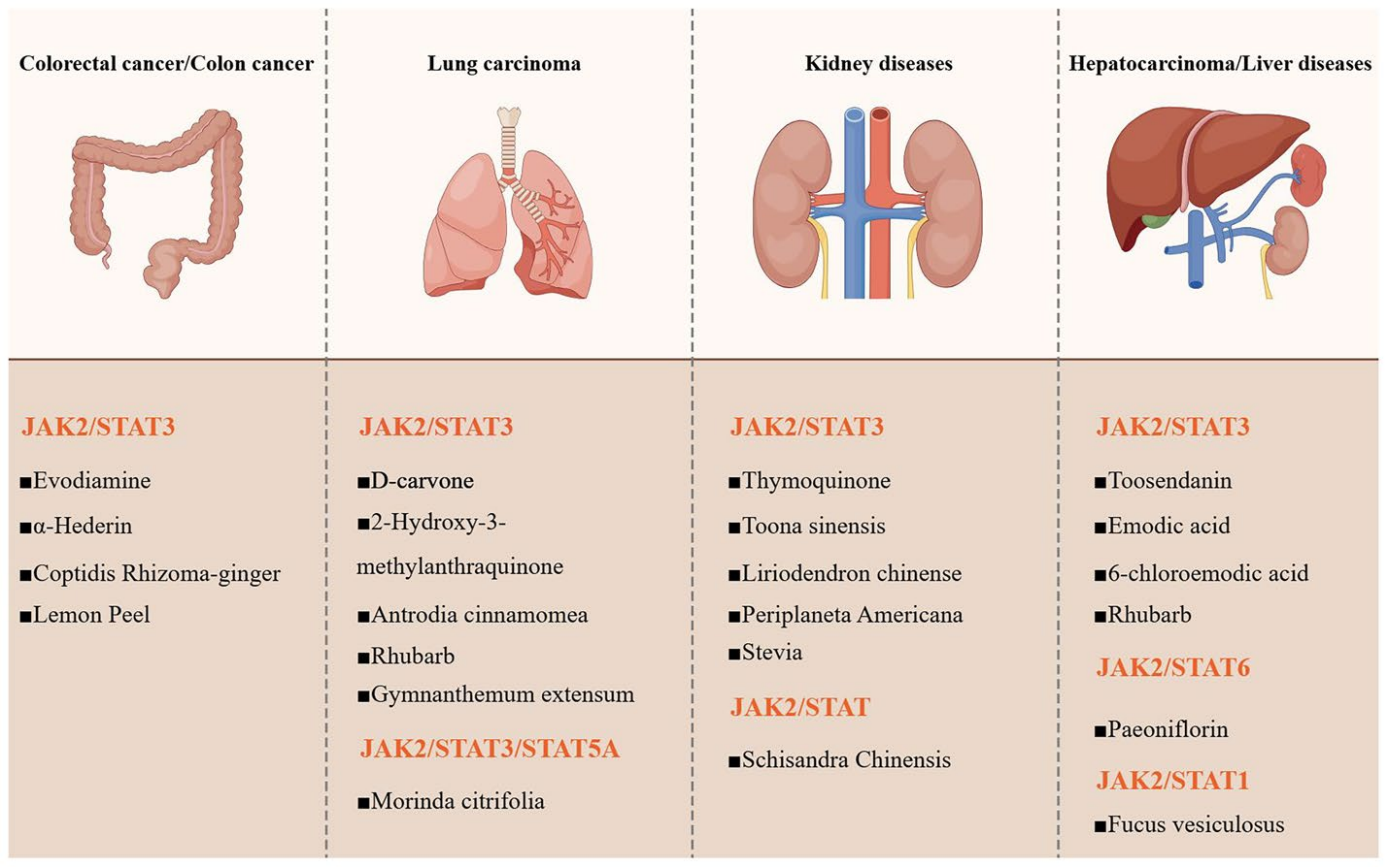
JAK2-associated diseases and potential therapeutic JAK2 inhibitors with signaling pathways. The four JAK2-associated diseases include colorectal cancer, lung carcinoma, kidney diseases and hepatocarcinoma. These pathologies share constitutive activation of the JAK2-STAT3 signaling axis. JAK, Janus kinase; STAT, signal transducer and activator of transcription

This study’s second phase identifies 32 potential JAK2 inhibitors with structurally characterized compounds (Table 3). These phytochemicals span six major classes: terpenes, polyphenols, phenylpropanoids, glycosides, alkaloids and quinones. Among these categories of natural compounds, polyphenols and terpenes constituted the largest proportion. Glycosides and quinones represent underrepresented classes in JAK1 inhibitor research. Therapeutic applications encompass esophageal cancer, osteosarcoma, NSCLC, colorectal cancer, HCC, depression and acute pancreatitis (Fig. 4).

**Table 3:** Potential JAK2 inhibitors with known chemical structures from natural products

Candidates CAS number	Chemical structures	Species	Classifications	IC_50_	Signaling pathways/Targets	Pharmacologic effects/Applications
D-carvone 140,698–12-0		*Carum carvi* L	Monoterpenes	< 100 μM	JAK2-STAT3; MAPK3	NSCLC
Sanshool 83,883–10-7		*Zanthoxylum bungeanum* Maxim	Monoterpenes	> 20 μM	AKT; JAK2-STAT3	Skin photodamage
Geniposide 27,745–20-6		*Gardenia jasminoides* J.Ellis	Iridoid glycoside	> 20 μM	BTK; JAK2-STAT1	Depression
Curcumol 4871–97-0		*Curcuma zedoaria* (Christm.) Roscoe	Sesquiterpenes	2.5–40 μg/L	JAK2-STAT3; PI3K-AKT; Wnt-β-Catenin	Endometriosis
2-desoxy-4β-propylcarbamate-pulchellin -		*Polygonum hydropiper* L	Sesquiterpenes	5–10 μM	JAK2-STAT3	Cancers
Toosendanin 58,812–37-6		*Melia azedarach* L	Triterpenes	< 0.12 μM	JAK2-STAT3; Wnt-β-Catenin	Hepatocellular carcinoma
α-arbutin 84,380–01-8		*Arctostaphylos uva-ursi* (L.) Spreng	Polyphenols	–	JAK2-STAT	Cancers; Central nervous system disorders; Osteoporosis; Diabetes
β-arbutin 497–76-7		*Arctostaphylos uva-ursi* (L.) Spreng	Polyphenols	< 100 μM	JAK2-STAT3	Cancers; Central nervous system disorders; Osteoporosis; diabetes
Phlorizin 60–81-1		*Lithocarpus polystachyus* (Wall. ex A.DC.) Rehder	Polyphenols	< 0.8 mM	JAK2-STAT3	Esophageal cancer
EGCG 989–51-5		*Camellia sinensis* (L.) Kuntze	Polyphenols	> 20 μM	JAK2-STAT3	Hypothalamic inflammation; Intestinal mucosal barrier protection; Vitiligo
Amentoflavone 1617–53-4		*Selaginella tamariscina* (P.Beauv.) Spring; *Selaginella rupestris* (L.) Spring; *Ginkgo biloba* L	Isoflavonoids	–	ERK; NF-κB; PI3K-AKT; Mpro; 3CLpro	Inflammatory responses; Oxidation; Microorganism infection; Metabolism regulation
Amentoflavone analogue 1 –		Selaginella tamariscina; Selaginella rupestris; Ginkgo biloba	Isoflavonoids	< 0.3 μM	JAK2-STAT3	Melanoma
Amentoflavone analogue 2 –		Selaginella tamariscina; Selaginella rupestris; Ginkgo biloba	Isoflavonoids	< 5 μM	JAK2-STAT	Melanoma
Baicalin 21,967–41-9		*Scutellaria baicalensis* Georgi	Isoflavonoids	–	B7H4; JAK2-STAT3; Nrf2-Keap1	Acute pancreatitis
5-Demethylnobiletin 2174–59-6		*Citrus reticulata* Blanco	Isoflavonoids	< 25 μg/mL	JAK2-STAT3	Inflammatory responses
Icariin 489–32-7		*Epimedium brevicornu* Maxim	Isoflavonoids	–	NF-κB; JAK2-STAT3	Acute lung injury
Farrerol 24,211–30-1		*Rhododendron dauricum* L	Isoflavonoids	< 25 μM	PIK3-AKT-mTOR; ERK; JAK2-STAT3	Angiogenesis-related diseases
Aspalathin 6027–43-6		*Aspalathus linearis* (Burm.f.) R.Dahlgren	Isoflavonoids	–	JAK2-STAT; PPARγ; SREBF1/2	Cardioprotection
Proanthocyanidin A1 103,883–03-0		*Litchi chinensis* Sonn	Isoflavonoids	–	JAK2-STAT3	Thrombocytopenia
Proanthocyanidin A2 41,743–41-3		*Litchi chinensis* Sonn	Isoflavonoids	< 50 μg/mL	JAK2-STAT3; PI3K-AKT-mTOR	Suppression of vascular endothelial growth factor
Psoralen 66–97-7		*Citrus limon* (L.) Burm. f.; *Raphanus sativus* L	Coumarins	< 30 μM	JAK2-STAT3; PI3K-AKT	Glioma
MC13 –		*Murraya paniculata* (L.) Jack	Coumarins	< 50 μM	TRAF6-TAK1-NF-κB; MAPK; ERK; JAK2-STAT1/3	Neuroinflammatory
Phillygenin 487–39-8		*Forsythia suspensa* (Thunb.) Vahl; *Forsythia koreana* (Rehder) Nakai	Glycosides	< 100 μM	JAK2-STAT3	Osteosarcoma
Paeoniflorin 23,180–57-6		*Paeonia* × *suffruticosa* Andrews; *Paeonia lactiflora* Pall.; *Paeonia veitchii* Lynch	Glycosides	–	AKT1; JAK2-STAT3/6	Type 2 diabetes mellitus
Fucoidan 9072–19-9		*Fucus vesiculosus* L.; *Sargassum kjellmanianum*; *Cladosiphon okamuranus* Tokida	Glycosides	–	JAK2-STAT1	Hepatic injury
Loganin 18,524–94-2		*Cornus officinalis* Siebold & Zucc	Glycosides	< 20 μM	JAK2-STAT3; Nrf2-HO-1; NF-κB	Cardiachpertrophy; Myocardial ischemia–reperfusion injury
Evodiamine 518–17-2		*Tetradium ruticarpum* (A.Juss.) T.G.Hartley	Alkaloids	> 50 μM	PGI; MMP3; JAK2-STAT3	Colorectal cancer
Rhynchophyllin 76–66-4		*Uncaria rhynchophylla* (Miq.) Miq	Alkaloids	–	JAK2-STAT3; NF-κB	Tourette syndrome
Thymoquinone 490–91-5		*Nigella sativa* L	Quinones	< 10 μM	JAK2-STAT3	Renal carcinoma
2-hydroxy-3-methylanthraquinone 17,241–40-6		*Hedyotis diffusa* Willd.; *Hedyotis corymbosa* (L.) Lam	Quinones	< 80 μM	JAK2-STAT3	Lung carcinoma
Emodic acid 478–45-5		*Xanthoria parietina*	Quinones	15 μM	JAK2-STAT3	Erythroleukemia
6-chloroemodic acid –		*Xanthoria parietina*	Quinones	10 μM	JAK2-STAT3	Erythroleukemia

### Terpenes

#### Monoterpenes

D-carvone (CN), a monoterpene primarily sourced from *Carum carvi* L., demonstrated JAK2-targeting potential in NSCLC [49]. A network pharmacology and molecular docking study indicated that CN may regulate JAK2 in NSCLC, with a reduction level of phosphorylation in H1299 and A549 cell lines [50]. Further experiments are anticipated to substantiate CN’s therapeutic efficacy.

Hydroxy-α-sanshool (Sanshool) is, the primary active ingredient in *Zanthoxylum bungeanum* Maxim [51]. Sanshool exhibited the photoprotective effects against UVB-induced damage in human dermal fibroblasts and animal models through suppressing the UVB-induced activation of JAK2-STAT3 signaling pathway [52]. These findings suggested sanshool as a promising candidate for protecting photodamaged skin.

#### Iridoid glycoside

Geniposide (GEN), derived from the *Gardenia jasminoides* J.Ellis, is an iridoid glycoside. A review summarized that GEN demonstrated multiple pharmacological activities, including hepatoprotective, anti-osteoporosis, antitumor and anti-diabetic effects [53]. Although GEN’s antidepressant potential has been largely overlooked, in vivo studies confirm its antidepressant activity via inhibition of BTK and JAK2-STAT1 pathway in LPS-induced depressive mice [54]. Further investigation into the antidepressant effects of GEN may provide valuable insights.

#### Sesquiterpenes

Curcumol, an active component from *Curcuma zedoaria* (Christm.) Roscoe, has exhibited antimicrobial, antioxidant, anti-inflammatory and anticancer properties [55]. Recent studies confirmed its anticancer efficacy against multiple solid tumors, including breast, colorectal, head and neck, and lung adenocarcinomas [56–58]. Specifically, Curcumol could inhibit the proliferation and migration of ectopic endometrial stromal cells and the phosphorylation of JAK2 and STAT3, thereby attenuating endometriosis [59]. This evidence strongly associates Curcumol with the JAK2-STAT3 pathway.

2-Desoxy-4β-propylcarbamate-pulchellin (P-13), a sesquiterpene lactone derivative of 2-desoxy-4-epi-pulchellin extracted from *Carpesium abrotanoides* L., has various pharmacological activities, including anti-inflammatory, antitumor, antiallergic, antioxidant, antithrombotic, antibacterial, myocardial protective and cerebral ischemia injury protective activities [60]. P-13 formed a covalent bond with JAK2, thereby inhibiting JAK2 with high efficacy. This revealed P-13 to be a novel therapeutic agent against cancer and further studies are anticipated to reveal additional bioactivities of P-13 [61].

#### Triterpenes

Toosendanin (TSN), belonging to triterpenoids, is the primary bioactive constituent of *Melia azedarach* L [62]. TSN downregulated the level of p-JAK2, leading to the upregulation of WWOX that may suppress the proliferation and metastasis of HCC cells [63]. Nevertheless, another study indicated that TSN-induced hepatotoxicity may stem from autophagy and lysosomal function inhibition via the STAT3-CTSC axis [64]. The mechanisms underlying TSN’s hepatocyte cytotoxicity require further clarification.

### Polyphenols

Arbutin, a bioactive polyphenol with α-isomer and β-isomer originating from *Arctostaphylos uva-ursi* (L.) Spreng, benefits the treatment of diseases, including various cancer, central nervous system disorders, osteoporosis and diabetes. And α-arbutin demonstrated to be more efficacious among isomers [65]. β-Arbutin ameliorated lead acetate-induced testicular injury and colitis through JAK2-STAT3 pathway inhibition [66, 67]. Further research is required to define the value of arbutin.

Phlorizin, the main constituent of *Lithocarpus polystachyus* (Wall. ex A.DC.) Rehder, is a dihydrochalcone. Previous studies have corroborated that phlorizin possesses a plethora of salutary effects, including antioxidant, anti-inflammatory, antimicrobial, cardioprotective, antidiabetic, and anticancer properties [68]. The JAK2-STAT3 pathway mediated Phlorizin’s inhibitory effects on the progress of esophageal cancer [69].

Epigallocatechin gallate (EGCG) is a potent bioactive component of *Camellia sinensis* (L.) Kuntze with diverse biological activities. Studies have illustrated that EGCG can attenuate hypothalamic inflammation, protect intestinal mucosal barrier function and decrease the risk of vitiligo by inhibiting the JAK2-STAT3 signaling pathways [70–72]. Therefore, we propose EGCG as a potential therapeutic adjuvant for multiple diseases.

#### Isoflavonoids

Amentoflavone (AMF), a natural bioflavonoid compound, has been isolated from several plants, including *Selaginella tamariscina* (P.Beauv.) Spring and *Ginkgo biloba* L. AMF exhibited multifunctional biological activities, such as anti-inflammatory activity, antimicrobial activity, pro-oxidative activity, neuroprotective activity and anti-cancer effects [73]. AMF has several structural analogs, two of which are discussed here. Virtual screening identified AMF1 as a potent non-competitive inhibitor of JAK2 and HCV, suggesting it may function as a Type II JAK2 inhibitor [74]. In contrast, AMF2 demonstrated a weaker activity in this screen. But in a separate study observed that AMF2 induces apoptosis in malignant melanoma cells as a JAK2 inhibitor [75]. Collectively, these findings suggest AMF2 possesses significant potential as a JAK2 inhibitor.

Baicalin (BI), a flavonoid extracted from the root of *Scutellaria baicalensis* Georgi, exert therapeutic effects in hepatobiliary and gastrointestinal disorders [61, 76, 77]. Studies indicated that BI inhibited the activation of B7H4 and JAK2-STAT3 signaling pathway, thereby reducing apoptosis and inflammation in hypertriglyceridemia-induced acute pancreatitis mice (HTG-AP) [78]. This finding elucidates the mechanism underlying BI’s therapeutic effects against HTG-AP.

5-Demethylnobiletin (5-DN) is predominantly extracted from *Citrus reticulata* Blanco, with pharmacological activities, including anti-inflammatory, antioxidant, antimicrobial, neuroprotection, anti-atherogenic effects [79]. The relationship between 5-DN and JAK2 remains underexplored. A study demonstrated that 5-DN repressed the expression of JAK2 and STAT3 in a neuroglia BV-2 Cell Line induced by LPS [80]. Further research is required to elucidate the neuroprotective mechanisms of 5-DN.

Icariin (ICA) is an active flavonoid component of *Epimedium brevicornu* Maxim with anti-inflammatory and anti-tumor activities [81, 82]. Research established that ICA inhibited tumor proliferation via the JAK2-STAT3 pathway. Furthermore, ICA ameliorated LPS-induced acute lung injury in mice by suppressing the same signaling pathways [83].

Farrerol, isolated from *Rhododendron dauricum* L, exerts vasoactive effect through JAK2-STAT3 inhibition [84]. Farrerol decreased the phosphorylation levels of JAK2 and STAT3 in HMEC-1 and HUVEC cells, and bound to the domain of STAT3 in docking assay [85]. These findings support Ferrero’s potential as a therapeutic agent for angiogenesis-related diseases.

Aspalathin, a C-glucosyl dihydrochalcone polyphenol isolated from *Aspalathus linearis* (Burm.f.) R.Dahlgren, demonstrated beneficial effects against inflammation, neurodegenerative disorders and Type 2 diabetes [86–88]. Transcription profile analysis showed that Aspalathin alleviated the lipid-induced inflammatory response via IL-6-JAK2-STAT pathway [86].

Proanthocyanidins (condensed tannins), polymeric flavan-3-ols with diverse pharmacological properties, are abundant in berries and fruits such as *Litchi chinensis* Sonn. Proanthocyanidin A1 and proanthocyanidin A2 are two bioactive isomers isolated by high-performance liquid chromatography [89]. The evidence indicated that due to their disparate spatial structures, they exhibited contrasting effects on the JAK-STAT signaling pathway. Proanthocyanidin A1 ameliorated chemotherapy-induced thrombocytopenia by activating JAK2-STAT3 signaling, enhancing JAK2 thermal stability and upregulating JAK2 expression [90]. Conversely, Proanthocyanidin A2 reduced NiCl_2_-induced VEGF expression in HepG2 cells through the inhibition of JAK2-STAT3 signaling pathway [91].

#### Coumarins

Psoralen, a natural furocoumarin in plants, such as *Citrus limon* (L.) Burm. f. and *Raphanus sativus* L., possess photosensitizing activity and anti-tumor properties. Upon ultraviolet radiation exposure, Psoralen penetrated epidermal cells to form DNA interstrand crosslinks, inducing cytotoxic effect that underpins its therapeutic application against severe psoriasis [92]. Moreover, Psoralen may suppress cell proliferation and migration, promote apoptosis and regulate the cell cycle arrest by inhibiting JAK2 [93]. However, its toxicities, including phototoxicity and hepatotoxicity, can’t be ignored. It’s necessary to determine how these toxic effects could be avoided.

MC13, a novel coumarin isolated from *Murraya paniculata* (L.) Jack has been demonstrated to inhibit the activation of the JAK2-STAT1/3 signaling pathway, thereby ameliorating neuroinflammation [94].

### Glycosides

Phillygenin (PHI), a bicyclic lignin compound, is initially isolated from *Forsythia suspensa* (Thunb.) Vahl and *Forsythia koreana* (Rehder) Nakai. As an intestinal metabolite of phillyrin, PHI has been demonstrated to possess a range of pharmacological effects, including anti-inflammatory, antioxidant, hepatoprotective, antitumor, antibacterial, antiviral, immunomodulatory, analgesic and anti-hypertensive activities [95, 96]. PHI inhibited osteosarcoma growth and metastasis by suppressing JAK2 and STAT3 phosphorylation [97]. And multiple studies indicated that PHI is a promising agent for malignant tumors, including HCC, pancreatic cancer, lung cancer and osteosarcoma [96–98].

Paeoniflorin, a monoterpenoid glycoside, is derived from *Paeonia* × *suffruticosa* Andrews, or Paeonia veitchii Lynch which distributed across temperate Eurasia, northwest Africa, and western North America [99]. Paeoniflorin’s positive effects have been identified on depression, malignant tumors and cardiovascular diseases [100–102]. Lili Zhang et.al. revealed that combined paeoniflorin-berberine (BBR + PF) therapy upregulated AKT1, JAK2 and STAT3 to mitigate type 2 diabetes mellitus [103]. Nevertheless, a previous research indicated that paeoniflorin exerted an inhibitory effect on alternative macrophages activation by modulating the JAK2-STAT6 signaling pathway [104]. These divergent mechanisms underscore the need for further target-specific elucidation of Paeoniflorin’s effects.

Fucoidan, a sulfated polysaccharide composed primarily of L-fucose and sulfate esters, has extended its application to renal disease [105]. Furthermore, several reviews summarized that fucoidan exhibited broad bioactivities, including anti-cancer, anti-inflammatory, anti-bacterial, anti-viral, neuroprotective and anti-HIV properties [106]. Fucoidan ameliorated IR-induced hepatic injury by blocking the release of upstream inflammatory factors of JAK2-STAT1 and decrease the phosphorylation levels of JAK2 and STAT1 [107].

Loganin, a monoterpene Iridoid glycoside isolated from *Cornus officinalis* Siebold & Zucc., showed diverse pharmacological effects, such as antidiabetic, anti-inflammatory, neuroprotective and antitumor properties [108]. Loganin’s effect on the JAK2-STAT3 pathway remains controversial. A study reported that Loganin protected against ischemia by downregulating JAK2-STAT3 and activating the Nrf2 signaling pathway [109]. Similarly, Loganin inhibited angiotensin II–induced cardiac hypertrophy through the suppression of the JAK2-STAT3 and NF-κB signaling pathways [110]. Conversely, another research reported that Loganin protected against myocardial ischemia–reperfusion injury by increasing the expression level of JAK2-STAT3 signaling [111]. Further investigations are required to resolve whether Loganin activates or suppresses this pathway.

### Alkaloids

Evodiamine (Evo), the primary alkaloid in *Tetradium ruticarpum* (A.Juss.) T.G.Hartley fruit, exhibited promising anti-cancer properties attributed to its distinctive L-shaped conformation [112, 113]. A study established that Evo induced apoptosis in human colorectal cancer cells by inactivating the JAK2-STAT3 pathway, as confirmed through rigorous apoptosis assays [114]. Thus, the JAK2-STAT3 pathway is mechanistically linked to Evo’s anti-tumor effects.

Rhynchophyllin, the primary bioactive ingredient of *Uncaria rhynchophylla* (Miq.) Miq., remains understudied despite extensive research on its source plant. Established evidence indicated that U. Rhynchophylla alleviated neurodegenerative diseases [115]. Leveraging this pharmacological property, Hongyan Long et.al found that Rhynchophylline attenuated neuroinflammation in a Tourette syndrome model via JAK2-STAT3 pathway [116].

### Quinones.

Thymoquinone (TQ), a principal bioactive component of *Nigella sativa* L., is regarded as a prospective anticancer agent [117]. Several reviews confirmed the TQ’s chemo preventive and anticancer activities across diverse malignancies, including breast, liver, colon, lung, renal carcinoma [117, 118]. A study indicated that TQ inhibited the JAK2-STAT3 pathway, thereby inducing apoptosis in human renal carcinoma Caki-1 cells [119]. Another study reported that TQ induced oxidative stress-mediated apoptosis through the inhibition of the JAK2-STAT3 signaling pathway in human melanoma cells [120]. The JAK2-STAT3 signaling pathway also involved in apoptosis in TQ induced apoptosis of K562 leukemia cells. Additionally, TQ enhanced antitumor efficacy when combined with gamma knife radiosurgery in B16-F10 melanoma through JAK2-STAT3 inhibition [121]. Collectively, JAK2-STAT3 regulation represented a mechanistically credible target for TQ-mediated tumor apoptosis.

2-hydroxy-3-methylanthraquinone (HMA), an anthraquinone monomer derived from *Hedyotis diffusa* Willd. and *Hedyotis corymbosa* (L.) Lam., serves as a chemical reference standard for *Hedyotis diffusa Willd* identification [122]. HMA demonstrated inhibitory effects against osteosarcoma and lung carcinoma [123]. And HMA was found to downregulate the IL-6-induced JAK2-STAT3 signaling pathway, thereby suppressing the growth and invasion of lung cancer cells [124].

Emodic acid and 6-chloroemodic acid emerged as potent JAK2 inhibitors through high-throughput virtual screening of a natural product database. Both compounds demonstrated dose-dependent inhibition of JAK2 activity in human erythroleukemia cells, confirming their biological efficacy [125].

### Total extracts

A collection of 70 different kinds of total extracts and their species, signaling pathways or targets, classifications of active ingredients, potential applications and references is represented (Table 4). We can find that most of the species are from traditional Chinese herbs and ingredients are commonly observed, such as *Rheum palmatum* L., *Vincetoxicum mukdenense* Kitag, *Angelica sinensis* (Oliv.) Diels and *Citrus* × *limon* (L.) Osbeck, *Toona sinensis* (A.Juss.) M.Roem., *Camellia sinensis* (L.) Kuntze. Consistent with TCM principles, these extracts leverage multicomponent synergism for therapeutic effects. Thus, TCM remains a vital source of mechanistic insights and drug discovery opportunities.

**Table 4:** Total extracts target JAK2

Species	Signaling pathways/targets	Classifications of active ingredients	Potential applications	Ref
*Acori Tatarinowii Rhizoma*	PI3K-AKT; MAPK; JAK2-STAT	–	Alzheimer's disease	[225]
*Agaricus blazei Murill*	JAK2-STAT3	–	Gastric cancer	[226]
*Alpinia katsumadae* Hayata	JAK2-STAT3	Acyclic triterpenes	Inflammatory responses	[227]
*Ampelopsis brevipedunculata* (Maxim.) Trautv	JAK2-STAT3; ERK	Glucosides	Inflammatory responses	[228]
*Angelica sinensis* (Oliv.) Diels (root)	JAK2-STAT1/3	Polysaccharides	Anemia; inflammatory responses	[229, 230]
*Antrodia cinnamomea*	JAK2-STAT3	–	Lung cancer	[231]
*Aruncus dioicus var.kamtschaticus*(dried aerial parts)	JAK2-STAT3; AKT-mTOR	–	Skin inflammation	[232]
*Bupleurum chinense* DC*.-Scutellaria baicalensis* Georgi	JAK2-STAT3; PI3K-AKT	–	Colorectal cancer	[233]
*Eremochloa ciliaris* (L.) Merr*.* (seeds)	JAK2-STAT3	Polyphenols	Aberrant immune responses	[234]
*Castanea mollissima* Blume (shell)	JAK2-STAT3	Polyphenols	Leptin-resistant obesity	[235]
*Cinnamomum zeylanicum* Blume	JAK2-STAT3; MMP-1; BcL-xL; Bax	–	Infliximab tolerance	[236]
*Citrus australasica* F.Muell*.* (fruits)	JAK2-STAT3; NF-κB; TLR	Polyphenols	Oxidant	[237]
*Citrus reticulata* Blanco (peel)	JAK2-STAT3; PI3K-AKT; MAPK	Flavonoids	Alleviating physical fatigue	[238]
*Clematis florida* Thunb	JAK2-STAT3	Saponins	Arthritis	[239]
*Coptis chinensis* Franch*.—Zingiber officinale* Roscoe	JAK2-STAT3; PI3K-AKT; SRC	–	Colon cancer	[240]
*Coreopsis tinctoria* Nutt*.* (flower)	JAK2-STAT; PI3K-AKT	Flavonoids	Diabetic	[241]
*Corydalis hendersonii* Hemsl	JAK2-STAT3; NF-κB	Alkaloids	Myocardial injury	[242]
*Vincetoxicum mukdenense* Kitag	JAK2-STAT; PI3K-AKT; MAPK	–	Bungarus multicinctus bites	[243]
*Rheum palmatum* L	MAPK; JAK2-STAT3	–	Acute pancreatitis; HCC	[244, 245]
*Eurycoma longifolia* Jack	JAK2-STAT3	–	Inflammatory responses	[246]
*Citrus maxima* (Burm.) Merr	JAK-STAT; MyD88; Nrf2-GPX4	Flavonoids	Acute lung injury	[247]
*Gardenia jasminoides* J.Ellis (air-dried gardenia fruits)	JAK2-STAT1	–	Focal cerebral ischemia; reperfusion injury	[248]
*Zanthoxylum bungeanum* Maxim	JAK2-STAT; AMPK; PI3K-AKT	Essential oil	Type 2 diabetes	[249]
*Fucus vesiculosus* L	JAK2-STAT1; TRADD-TRAF2	Polysaccharides	Acute liver injury	[250]
*Ganoderma lucidum* (Curtis) P. Karst	JAK2-STAT	–	Hyperglycemia	[251]
*Garcinia xanthochymus* Hook.f. ex T.Anderson (fruits)	JAK2-STAT3	Polyphenols	Tumor	[252]
*Geum aleppicum* Jacq*.* (entire plant)	JAK2-STAT3; PI3K-AKT	–	Hematopoietic function	[253]
*Ginkgo biloba* L	JAK2-STAT3; MAPK; NF-κB; Wnt-β-catenin	–	Inflammatory responses; apoptosis	[254]
*Gymnanthemum extensum* (DC.) Steetz	JAK2-STAT3	Sesquiterpene Lactones	Lung carcinoma	[255]
*Hordei Fructus germinatus*	JAK2-STAT5	–	Prolactin	[256]
*Hovenia dulcis* Thunb. (fruits)	MAPK; AP-1; JAK2-STAT; NF-κB	Flavonoids	Inflammatory responses	[257]
*Trametes robiniophila* Μurr	JAK2-STAT3; MAPK	–	Tuberous sclerosis	[258]
*Humulus scandens (Lour.)* Merr	JAK2-STAT5	–	Longitudinal bone growth	[259]
*Ilex asprella* (Hook. & Arn.) Champ. ex Benth	NF-κB; JAK2-STAT3; MAPK	–	Inflammatory responses	[260]
*Lagopsis supina* (Steph. ex Willd.) Ikonn*.—Gal*	JAK2-STAT3	–	Colorectal cancer	[261]
*Citrus* × *limon* (L.) Osbeck (Peel)	JAK2-STAT3	Polyphenols	Colon cancer	[262]
*Liriodendron chinense* (Hemsl.) Sarg*.* (barks)	NF-κB; ASK1-JNK; JAK2-STAT3	–	Inflammatory responses	[263]
*Litsea cubeba* (Lour.) Pers	JAK2-STAT3; NF-κB	Alkaloids	Inflammatory responses	[264]
*Camellia sinensis* (L.) Kuntze	JAK2-STAT3	–	Hypothalamic inflammation	[265]
*Morinda citrifolia* L*.* (leaves)	BIRC5; JAK2-STAT3	Polyphenols	Metastasized lung cancer	[266]
*Mucuna pruriens* (L.) DC*.* (seeds)	JAK2-STAT5A	L-Dopa	Breast cancer	[267]
*Muntingia calabura* L*.* (fruit)	JAK2-STAT1/3; NF-κB; MAPK	Flavonoids	Inflammatory responses	[268]
*Natsiatum herpeticum* Buch. (aerial parts)	JAK2-STAT3; EGFR; PPARG; PTGER4; PPARA	–	Inflammatory responses	[269]
*Nervilia fordii* (Hance) Schltr	JAK2-STAT3	Flavonoids	Polycystic ovary syndrome	[270]
*Origanum majorana* L. (flowers)	JAK2-STAT3; NF-κB	–	Colitis	[271]
*Paeonia lactiflora* Pall. (dry root)	TGF-β-SMAD; PI3K-AKT; JAK2-STAT3	–	Hepatic fibrosis	[272]
*Periplaneta Americana*	JAK2-STAT3	–	Renal fibrosis	[273]
*Colla Apis*	JAK2-STAT3; NF-κB	–	Virus infection	[274]
*Rhamnella gilgitica* Mansf. & Melch	JAK2-STAT3	Flavonoids	RA	[275]
*Rheum palmatum* L	EGFR; BCL2; JAK2-STAT	Polyphenols	NSCLC	[276]
*Rubus chingii* Hu	JAK2-STAT1/3	Flavonoids	Macrophage activation	[277]
*Carthamus tinctorius* L	JAK2-STAT3	Flavonoids	Parkinson's disease	[278]
*Salvia miltiorrhiza* Bunge	JAK2-STAT3	Quinones	Acute myeloid leukemia; acute pancreatitis	[244, 279]
*Salvia plebeia* R. Br*.* (aerial parts)	JAK2-STAT3; MAPK	–	Bone loss	[280]
*Sanguisorba officinalis* L	JAK2-STAT1	–	Inflammatory responses	[281]
*Schisandra chinensis* (Turcz.) Baill	PI3K-AKT; VEGFA; NOS3; JAK2-STAT	–	Diabetic nephropathy	[282]
*Hippophae rhamnoides* L*.* (seeds, berry flesh, and peel)	NF-κB; JAK2-STAT1; MAPK	Essential oil	Atopic dermatitis	[283]
*Citrus depressa* Hayata (fruit)	Caspase 3; JAK2-STAT	Flavonoids	Osteoarthritis	[284]
*Spatholobus suberectus* Dunn	JAK2-STAT5	–	Hematopoietic alteration; oxidative stress	[285]
*Stevia rebaudiana* (Bertoni) Bertoni (residue)	JAK2-STAT3; Nrf2	Terpenoids	Renal injury	[286]
*Toona sinensis* (A.Juss.) M.Roem*.* (Tender leaves)	JAK2-STAT3; MEK-ERK; mTOR-HIF-2α	–	Renal Carcinoma	[287]
*Toxicodendron vernicifluum* (Stokes) F.A.Barkley (dried heartwood powder)	PI3K-AKT-mToR-Gsk3β; JAK2-STAT3; MAPK	Flavonoids	Oxidative stress	[288]
*Tribulus terrestris* L*.* (fruits)	JAK2-STAT3; PI3K-AKT	–	Endothelial dysfunction; hypertensive endothelial injury	[289, 290]
*Uncaria rhynchophylla* (Miq.) Miq. ex Havil*. -Eucommia ulmoides* Oliv	AKT1; NOS2; ADRB2; JAK2-STAT	–	Pregnancy hypertension	[291]
*Veronica polita subsp. polita*	JAK2-STAT3; NF-κB	Flavonoids; polyphenols	Murine colitis	[292]
*Chrysopogon zizanioides* (L.) Roberty	JAK2-STAT3; ERK1/2	–	RA	[293]
*α-Hederin*	JAK2-STAT3	Triterpenoid saponins	Colon cancer	[294]

The biflavonoid skeleton demonstrates significant potential in the activity of JAK2 inhibitors, which is evidenced by the substantially higher inhibitory activity of amentoflavone analogues compared to EGCG. Certain terpenoids, such as Toosendanin, Curcumol, and Pulchellin, also exhibit excellent activity. Chlorine substitution on the benzene ring of quinone compounds can enhance inhibitory activity to some extent, as in the case of 6-chloroemodic acid displaying a lower IC_50_ value than Emodic acid. Hydroxyl groups are prevalent in natural JAK2 inhibitors, and their substitution position influences inhibitor potency.

## Potential JAK3 inhibitors from natural products

JAK3 represents a promising therapeutic target for hematological malignancies, such as leukemic, β-thalassemia [126, 127]. In addition, JAK3 inhibitors, MJ40 and *Lagerstroemia indica* L. extract, exhibited efficacy against hair loss [128, 129]. Therefore, natural product-derived JAK3 inhibitors hold significant potential for treating both hematologic disorders and hair loss. Potential JAK3 inhibitors with known structures are presented (Table 5).

**Table 5:** Potential JAK3 inhibitors with known chemical structures from natural products

Candidates CAS number	Chemical structure	Species	Classifications	IC_50_	Signaling pathways/Targets	Pharmacologic effects/applications
Dihydroarteannuin 71,939–50-9		*Artemisia annua* L	Sesquiterpenes	< 0.4 μM	JAK3-STAT3; HIF-1α	Arthritis
Panaxadiol 19,666–76-3		*Panax ginseng* C.A.Mey	Triterpenoids	< 8 μM	JAK3-STAT3	Cerebral ischemic stroke
Icaritin 118,525–40-9		*Epimedium brevicornu* Maxim	Flavonoids	6.25–12.5 μM	JAK3-STAT5	Inflammatory responses; immunomodulation
Atractylenolide III 73,030–71-4		*Atractylodes macrocephala* Koidz	Lactones	< 16 μM	JAK3-STAT3	Lung cancer
Stigmasterol 83–48-7		*Glycine max* (L.) Merr	Steroids	< 5 μM	JAK3-STAT	Breast cancer
Cryptotanshinone 35,825–57-1		*Salvia przewalskii* Maxim; *Salvia tebesana* Bge	Anthraquinones	6.25 μM	JAK3-STAT5	Cardiovascular diseases; inflammatory responses
Astilbin 29,838–67-3		*Smilax glabra* Roxb	Glycosides	–	JAK3-STAT3	Psoriasis
MJ04 –		3-pyrimidinylazaindole	Alkaloids	2.03 nM	JAK3-STAT	Hair loss
Indirubin 479–41-4		*Strobilanthes cusia* (Nees) Kuntze	Alkaloids	6.25 μM	JAK3-STAT5	Cancer, inflammatory responses; neuroprotective properties

### Terpenes

#### Sesquiterpenes

Dihydroarteannuin (DHA), primary element of artemisinin extracted from *Artemisia annua* L., exhibits potent anti-RA activity. A recent study showed that DHA exhibited significant therapeutic effects on arthritis by reducing HIF-1α expression and the phosphorylation of JAK3 and STAT3 [130]. Therefore, DHA is regarded as a potential therapeutic agent for RA treatment.

#### Triterpenoids

Panaxadiol, isolated from *Panax ginseng* C.A.Mey., belongs to triterpenoid saponin compounds. Research showed that Panaxadiol inhibited neuronal apoptosis by modulating the JAK3-STAT3-HIF-1α signaling pathway, thereby alleviating cerebral ischemic stroke [131].

### Lactones

Atractylenolide III is the primary active product of *Atractylodes macrocephala* Koidz., with limited prior reports on its anticancer effects through modulating the immune microenvironment. A study revealed that Atractylenolide III suppressed the activation of Indoleamine 2,3-dioxygenase-1 (IDO) by directly binding to JAK3 [132]. In summary, Atractylenolide III could regulate the tumor microenvironment in lung cancer, offering a novel target for immunotherapy of lung cancer.

### Glycosides

Astilbin, a major active flavonoid component of the rhizome of *Smilax glabra* Roxb., has potential for application in inflammatory diseases. A mechanistic study revealed that astilbin suppressed Th17 cell differentiation by inhibiting the JAK3-STAT3 signaling pathway, thereby improving psoriasis symptoms [133]. Additionally, astilbin demonstrated favorable safety and therapeutic efficacy in clinical models.

### Steroids

Stigmasterol, a common phytosterol rich in Glycine max (L.) Merr., attracted attention due to anti-cancer property. A study genetically demonstrated that stigmasterol reduced the characteristics of triple-negative breast cancer stem cells by inhibiting JAK3 [134]. The promising anti-tumor efficacy and favorable safety profile of stigmasterol suggest its potential as a novel therapeutic approach for breast cancer treatment.

### Alkaloids

MJ04 is a highly potent and selective JAK3 inhibitor based on 3-pyrimidinylazaindole scaffold series of compounds. Molecular docking, in vitro, and in vivo experiments have demonstrated its JAK3 inhibitory activity [128]. Furthermore, MJ40 also exhibited promising safety and pharmacokinetic properties, supporting its potential development as a therapeutic agent.

Indirubin, a bis-indole alkaloid derived from *Strobilanthes cusia* (Nees) Kuntze, alleviated psoriasis by suppressing γδ T cell-mediated inflammatory responses through inhibition of the JAK3-STAT3 signaling pathway [135]. Its selective inhibition of JAK3 is further substantiated in another independent study [136].

### Total extracts

Literature analysis identifies multiple plant extracts exhibiting JAK3 inhibitory activity, including *Polyphaga plancyi* [137, 138], *Calyptranthes grandifolia* O.Berg [126], *Lagerstroemia indica* L [129], *Boesenbergia rotunda (L.)* Mansf [139], *Ceiba speciosa* (A.St.-Hil.) Ravenna [140] and *Garcinia species* [141]. While active constituents remain uncharacterized, these findings establish critical research vectors for discovering novel JAK3 inhibitors. Additionally, documented natural compounds—cryptotanshinone, icaritin, and indirubin—showed potential as JAK3 kinase inhibitors [142].

Compounds with planar heterocyclic rings (such as MJ04 and Indirubin) demonstrate superior inhibitory potential against JAK3. This suggests molecules possessing certain planarity and rigidity may bind more readily to the JAK3 active pocket. Several terpenoids, flavonoids, and steroids (such as Dihydroarteannuin, Icaritin, and Stigmasterol) also exhibit moderate inhibitory activity against JAK3. Additionally, appropriate molecular weight and hydrophobic structures are essential characteristics for JAK3 inhibitors.

## Potential TYK2 inhibitors from natural products

TYK2 associates with immune cytokines subunits, playing a significant role in autoimmune and inflammatory diseases, including RA, inflammatory bowel diseases, psoriasis [143–145]. Currently, Deucravacitinib, as the first approved selective TYK2 inhibitor, has been used to treat psoriasis. This enlightens us that targeting TYK2 to develop related therapeutic drugs is feasible. But due to insufficient attention paid to TYK2 in the past, there are only several reports on TYK2 inhibitors from natural products (Table 6).

**Table 6:** Potential TYK2 inhibitors with known chemical structures from natural products

Candidates CAS number	Chemical structures	Species	Classifications	IC_50_	Signaling pathways/targets	Pharmacological effects/applications
β-caryophyllene 87–44-5		*Cannabis sativa* L.; *Neolitsea cassia* (L.) Kosterm.; *Syzygium aromaticum* (L.) Merr. & L.M.Perry; *Origanum vulgare* L; *Piper nigrum* L	Sesquiterpenes	–	IL-2; IL-6; IRF7; NLRP3; TYK2	Inflammatory responses; Tumor; Bactericidal properties
Oleanolic acid 508–02-1		*Swertia mileensis* T.N.Ho & W.L.Shih	Triterpenoids	> 25 μM	TYK2-STAT1/3; SOCS3	Obesity
Cannabidiol 13,956–29-1		*Cannabis sativa* L	Polyphenols	< 5 μM	NF-κB; TYK2-STAT3	Cytokine Storm
Δ9-tetrahydrocannabinol 1972–08-3		*Cannabis sativa* L	Polyphenols	< 5 μM	NF-κB; TYK2-STAT3	Cytokine Storm
Smeathxanthone A –		*Garcinia mangostana* L	Flavonoids	-	TYK2; MAPK14; ACE	COVID-19
Trapezifolixanthone 50,816–23-4		*Garcinia mangostana* L	Flavonoids	-	TYK2; MAPK14; ACE	COVID-19
E738 -		*Indigofera tinctoria* L	Alkaloids	0.7 nM	TYK2; SFKs	Pancreatic cancer
Docosahexaenoic acid 81,926–94-5		*Dasyatis akajei*(Muller et Henle)	Polyunsaturated fatty acids	–	IL-2; IL-6; IRF7; NLRP3; TYK2	Brain health diseases

### Terpenes

#### Sesquiterpenes

β-Caryophyllene is a natural sesquiterpene compound extracted from *Cannabis sativa* L.; *Neolitsea cassia* (L.) Kosterm.; *Syzygium aromaticum* (L.) Merr. & L.M.Perry; *Origanum vulgare* L.; and *Piper nigrum* L. A study have shown that β-caryophyllene could synergize with polyunsaturated fatty acids such as docosahexaenoic acid to exert potent anti-inflammatory and anti-nociceptive effects. This bioactivity is associated with the negative regulation of gene expression involved in intracellular inflammatory signaling cascades, including IL-2, IL-6, IRF7, NLRP3, and TYK2 [146].

#### Triterpenoids

Oleanolic acid, a triterpenoid compound extracted from *Swertia mileensis* T.N.Ho & W.L.Shih, possesses antioxidant and anti-aging properties. A study indicated that oleanolic acid inhibited resistin production associated with adipocyte differentiation by interfering with the TYK2-STAT1/3 signaling pathway and promoting SOCS3 expression [147]. This suggests oleanolic acid may benefit weight loss in obese patients.

### Polyphenols

Cannabidiol and Δ9-tetrahydrocannabinol are the primary active components of *Cannabis sativa* L., extensively studied for their potent anti-inflammatory effects. A study demonstrated that cannabidiol and Δ9-tetrahydrocannabinol significantly reduced the LPS-induced increasement in TYK2 expression but had no effect on normal TYK2 expression [148]. This may be attributed to their influence on the production of the upstream cytokine IL-6.

#### Flavones

Smeathxanthone A and Trapezifolixanthone are two flavonoids extracted from *Garcinia mangostana* L., which exhibited strong binding affinity toward TYK2 in molecular docking simulations [149]. The inhibitory effects of these two flavonoids on TYK2 still require confirmation through molecular experiments, and whether they exhibit inhibitory activity against JAK kinases of the same family remains unknown.

### Alkaloids

E738 is a derivative of Indirubin. After structural modification, E738 exhibits highly selective inhibitory activity against TYK2. Additionally, E738 could also be used for the treatment of human pancreatic cancer by inhibiting the JAK2/Src-STAT3 signaling pathway [150].

Indole alkaloid (E738) exhibits the strongest known inhibitory activity against TYK2, likely due to its indole ring serving as a rigid planar structure that facilitates entry into the TYK2 active pocket. The carbonyl and hydroxyl substitutions, along with the N-heteroatom, optimize hydrogen bonding and ionic interactions with the hydrophobic region of TYK2. Therefore, hydrogen bond donors/acceptors and hydrophobic regions are critically involved in binding to TYK2.

### Total extracts

The number of traditional Chinese medicines targeting the TYK2 protein is extremely limited. Based on the origins of the above different categories of TYK2-targeting compounds, we speculate that herbal medicines such as Cannabis sativa L., Garcinia mangostana L., and Indigofera tinctoria L. may exhibit selective inhibitory effects on TYK2. Simultaneously, Plants extracts from *Citrus* × *limon* (L.) Osbeck, *Isatis tinctoria* L. increased the expression of TYK2, exhibiting anti-cancer and antiviral potential [151, 152].

## Potential multi-target inhibitors from natural products

JAK family members share extensive homologous domains, which leads to pan-inhibitory activity of many natural compounds against JAKs, making it extremely challenging to discover inhibitors targeting specific JAK members. However, subtle differences exist in the three-dimensional structures among JAK family members. Although different natural compounds exhibit pan-inhibitory activity, they demonstrate varying inhibitory potency against distinct JAK kinases. These characteristics will help guide the modification of these natural compounds to enhance their selectivity, providing significant value for directing drug development.

**Fig. 5:**
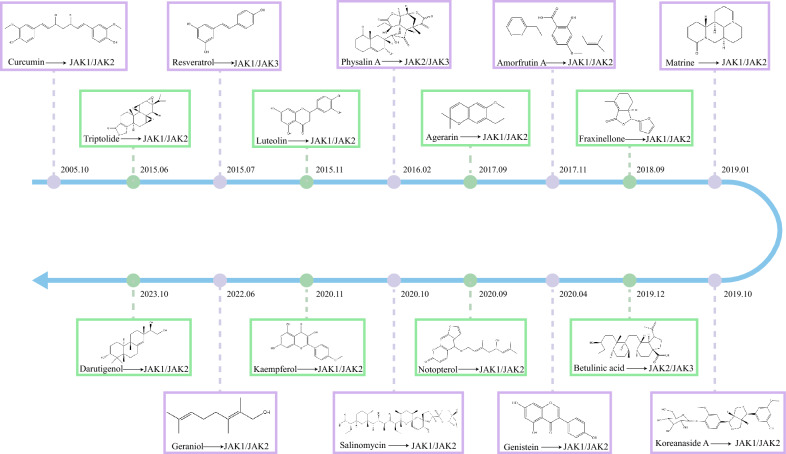
Potential multi-targets inhibitors with their targets. The chemical structures of the 17 compounds and their targets of action are shown on the axes according to the chronological order of the compounds in the research field of targeting JAK kinases. JAK, Janus kinase

A collection of 20 natural multi-target inhibitors with known structures is present (Table 7). These natural compounds span diverse chemical classes, including terpenes, polyphenols, phenylpropanoids, steroids, polyethers and alkaloids. Most concurrently modulate two distinct JAK kinase classes, enabling broad therapeutic applicability beyond single-target inhibition. Below we present the targets of these natural products (Fig. 5).

**Table 7:** Potential multi-targets inhibitors with known chemical structures from natural products

Candidates CAS number	Chemical structures	Species	Classifications	IC_50_	Signaling pathways/Targets	Pharmacologic effects/applications
Geraniol 106–24-1		*Lavandula angustifolia* Mill	Monoterpenes	–	CHRM3; PRKCA; PRKCD; JAK1/2	Alzheimer’s disease
3-O-methylthespesilactam -		Thespesilactam	Sesquiterpenes	JAK1:1.80 μM; TYK2:2.72 μM	JAK1/TYK2-STAT3; JAK2/3-STAT3	Melanoma
Triptolide 38,748–32-2		*Tripterygium wilfordii* Hook F	Diterpenes	JAK1: < 100 nM; JAK2: < 100 nM	EGFR; JAK1/2-STAT1/3	Ankylosing spondylitis; NSCLC
Darutigenol 5940–00-1		*Sigesbeckia orientalis* L	Diterpenes	–	JAK-STAT3	Arthritis
Crocin 42,553–65-1		*Crocus sativus* L	Diterpenes	JAK1: 10–20 μM; JAK2: 10–20 μM	JAK1/2/Src-STAT3	Multiple myeloma cells
Betulinic acid 472–15-1		*Acacia auriculiformis* Benth	Triterpenes	–	JAK2/3-STAT3; ABL1; GSK-3α/β	Leukemic
Curcumin 458–37-7		*Curcuma longa* L	Polyphenols	JAK1: 20 μM; JAK2: < 50 μM	JAK2-STAT3; JAK1-STAT5	Acute myeloid leukemia; Colitis; Primary effusion lymphoma growing
Resveratrol 501–36-0		*Curcuma longa* L.; *Arachis hypogaea* L	Polyphenols	JAK1: < 30 μM; JAK2: < 50 μM	JAK1/3-STAT5; JAK1/2/TYK2-STAT3/5	T-cell acute lymphoblastic leukemia; Myeloproliferative neoplasms; RCC
Piceatannol 10,083–24-6		*Curcuma longa* L.; *Arachis hypogaea* L	Polyphenols	JAK1: < 20 μM	JAK1-STAT1/3	Atopic dermatitis
Kaempferol 520–18-3		*Kaempferia galanga* L	Polyphenols	–	JAK1/2/Src-STAT3; SHP-1	Atopic dermatitis; Pancreatic cancer
Luteolin 491–70-3		*Reseda odorata* L	Flavonoids	JAK1: < 12.5 μg/mL	JAK1-STAT6; SOCS1; JAK2-STAT3	Ulcerative colitis; Atopic dermatitis
Genistein 446–72-0		*Glycine max* (L.) Merr	Flavonoids	JAK2: < 40 μM	JAK1/2-STAT3; SOCS3	Esophageal-carcinoma; Ulcerative colitis; Liver fibrosis
Amorfrutin A 80,489–90-3		*Amorpha fruticosa* L	Flavonoids	JAK1: < 40 μM; JAK2: < 40 μM	JAK1/2/Src-STAT3	Cervical cancer; Colon cancer; Breast cancer
Agerarin –		*Ageratum houstonianum* Mill	Chromenes	JAK1: 0.473 μM; JAK2: 4.92 μM; JAK3: 3.12 μM	JAK1/2 -STAT3	Atopic dermatitis
Fraxinellone 28,808–62-0		*Dictamnus dasycarpus* Turcz	Furanones	JAK1: < 30 μM; JAK2: < 30 μM	JAK1/2/Scr-STAT3	Tumor
Notopterol 88,206–46-6		*Hansenia weberbaueriana* (Fedde ex H.Wolff) Pimenov & Kljuykov	Coumarins	JAK1: > 2000 μM; JAK2:9.51 μM; JAK3:25.2 μM; TYK2:667 μM	JAK2/3-STAT3; NF-κB	Arthritis
Koreanaside A –		*Forsythia koreana* (Rehder) Nakai	Lignans	JAK1: < 40 μM; JAK2: < 40 μM	JAK1/2- STAT1/3; NF-κB	Colitis
Physalin A 23,027–91-0		*Alkekengi officinarum var. franchetii* (Mast.) R.J.Wang	Steroids	JAK2: < 5 μM; JAK3: < 5 μM	JAK2/3-STAT3	NSCLC; HCC
Salinomycin 53,003–10-4		*Streptomyces albus*	Polyethers	JAK1: < 10 μM; JAK2: < 10 μM	JAK1/2-STAT1/3	Breast cancer
Matrine 519–02-8		*Sophora flavescens* Aiton	Alkaloids	–	JAK2-STAT3	Cholangiocarcinoma

### Terpenes

#### Monoterpenes

Geraniol, a monoterpene alcohol with a pleasant aroma, is widely distributed in the *Fabaceae* family. Researches have shown that geraniol had varieties of pharmacological activities, such as anti-inflammatory, anticancer, antimicrobial, antioxidant, and neuroprotective activities [153–155]. Alzheimer’s disease (AD), a neurodegenerative disease with incompletely understood pathogenesis, may involve JAK1 and JAK2 as therapeutic targets of geraniol [156]. Further investigation is required to establish geraniol’s therapeutic potential for AD.

#### Sesquiterpenes

3-O-methylthespesilactam, a 3-O-methyl derivative of thespesilactam, was identified as a novel class of anticancer sesquiterpenes targeting JAK in A2058 human melanoma cells. 3-O-methylthespesilactam inhibited cell viability in human cancer cells and induced apoptosis and S-phase cell-cycle arrest in A2058 melanoma cells [157]. These findings suggest that 3-O-methylthespesilactam may represent a promising lead compound against human cancer cells.

#### Diterpenes

Triptolide (TPL), a bioactive diterpene triperoxide isolated from *Tripterygium wilfordii*. demonstrated anti-inflammatory, immunosuppressive, and antitumor properties [158]. JAK1 expression levels correlated with TPL’s cytotoxic response [159]. Meanwhile, JAK2 was the target of TPL intervention CTD-ILD and TPL could restrain the activation of the JAK2-STAT3 signaling pathway in ankylosing spondylitis [160, 161]. In terms of tumor immunity, TPL reduced PD-L1 expression through the EGFR and JAK1/2-STAT1/3 signaling pathways in NSCLC cells [12].

Darutigenol (DL), a diterpenoid derived *Sigesbeckia orientalis* L., exhibited anti-inflammatory activity [162, 163]. DL ameliorated inflammation and cartilage degradation in murine arthritis models via inhibition of JAK-STAT3 pathway, as evidenced through integrated network pharmacology [164].

Crocin, a bioactive constituent of *Crocus sativus* L., has been applied as an anodyne, aphrodisiac, and emmenagogue. A study demonstrated that crocin can mediate the suppression of STAT3 and inhibit the upstream kinases JAK1, JAK2, and SRC, thereby preventing the progression of multiple myeloma [14].

#### Triterpenes

Betulinic acid (BA), a natural pentacyclic triterpene compound, is isolated from *Acacia auriculiformis* Benth. with anti-inflammatory, antibacterial, antidiabetic, anti-HIV and antitumor effects [165, 166]. Study showed that BA could mitigate T-2 toxin-induced testicular injury by reducing germ cell apoptosis through JAK2-STAT3 signaling downregulation [167]. Betulin, the precursor of betulinic acid, could inhibit JAK3 conferring significant antitumor potential [168].

### Polyphenols

Curcumin (diferuloylmethane), a natural yellow polyphenol pigments isolated from the rhizomes of *Curcuma longa* L., had a wide range of applications, such as playing a synergistic effect on Alzheimer’s disease, antiviral effect, intestinal mucosal barrier function protective effect, anticancer effect [169–172]. Curcumin mitigated acute myeloid leukemia by upregulating p53 pathway and downregulating the JAK2-STAT3 pathway [173]. Curcumin targeted JAK1 to ameliorate dextran sulfate sodium-induced colitis and inhibit primary effusion lymphoma growth [171, 172].

Resveratrol, a naturally occurring polyphenol abundant in grapes, peanuts, red wine and certain plants, exhibits anticancer activity against T-cell acute lymphoblastic leukemia [174], myeloproliferative neoplasms [175], renal cell carcinoma [176] through JAK1and JAK2 inhibition. Moreover, its anti-inflammation effects extended to RAW 264.7 macrophages activated microglia cells and BTBR T autistic mouse models [177–179]. Piceatannol, the metabolite of Resveratrol, attenuated AD by targeting JAK1 and showed higher therapeutic efficacy than resveratrol [180]. Learned from this, resveratrol has a great potential for structural modifications through which resveratrol improves bio-targeting itself.

Kaempferol, a natural plant flavonoid compound is isolated both from *kaempferia galanga* L. A TCM composite formula, Jiu-Wei-Yong-An formula containing Kaempferol, alleviated AD-like skin lesions through suppressing JAK1-STAT3 and MAPK signaling pathways [181]. And Kaempferide (KF), a Kaempferol derivative, dose-dependently decreased the phosphorylation of JAK1, Src and STAT3 in the pancreatic cancer cell lines [158]. Besides, kaempferol downregulated the JAK1-STAT3 signaling pathway by which it inhibited the activation of neutrophils in peripheral blood and their infiltration into the ischemic brain. Paradoxically, Shanshan Zhang et al. reported that kaempferol didn’t inhibit JAK2 in neutrophils. In another research, Kaempferol promoted glucose uptake in myotubes via JAK2 inhibition and was considered as an effective compound for the prevention of hyperglycemia [182, 183]. Whether Kaempferol targeting JAK2 is cell-specific needs further validation.

#### Flavones

Luteolin (LUT), a flavonoid polyphenolic compound ubiquitously present in fruits, vegetables, flowers, and herbs. demonstrated anti-inflammatory properties through JAK-STAT pathway modulation. Luteolin-7-O-glucoside alleviated dextran sodium sulfate-induced ulcerative colitis mice by decreasing the secretion of inflammatory factors and reducing inflammatory responses via the JAK1-STAT6-SOCS1 pathway [184]. Similarly, this pathway also underpinned LUT’s efficacy against long-COVID inflammatory sequelae [185]. JAK2-STAT3 is possibly another signaling pathway for LUT’s anti-inflammatory property. Combining network pharmacology and experimental validation, Tang Liu et.al confirmed that LUT alleviated AD by inhibiting the JAK2-STAT3 signaling [19].

Genistein, a phytoestrogen abundant in *Glycine max (L.)* Merr., showed its diverse bioactivities, including anti-inflammatory, antioxidant, anticancer properties [186–188]. JAK-STAT pathway constitutes a key mechanism underlying these effects. Researches revealed that genistein inhibited the proliferation of esophageal-carcinoma cell and ameliorated acetic acid-induced ulcerative colitis via JAK1/2-STAT3 suppression. Moreover, genistein exhibited hepatoprotective effect on dimethyl nitrosamine induced liver fibrosis models by inhibiting the JAK2-STAT3-SOCS3 signaling pathway [189]. We could conclude that JAK2-STAT3 played an important role in Genistein’s biological activities.

Amorfrutin A, a natural product isolated from the fruits of *Amorpha fruticosa* L., exhibits documented anti-inflammatory properties through PPARα/γ agonism [190, 191]. But the mechanism of anticancer activity of Amorfrutin A was associated with JAK-STAT signaling [192].

#### Chromenes

Agerarin, is a bioactive compound derived from the ethanolic extract of *Ageratum houstonianum* Mill [193]. Agerarin abrogated IL-4-induced PER2 expression in HaCaT Cells through the JAK-STAT3 signaling pathway and ameliorated skin inflammation especially AD [194].

### Furanones

Fraxinellone (FRA), a degraded limonoid isolated from the root bark of *Dictamnus dasycarpus* Turcz., belongs to furanone compounds in chemical structure. FRA had potent insecticidal activity and pro-apoptosis effects in tumor cells [195]. A study revealed that FRA may inhibit PD-L1 expression via JAK1/2/Scr-STAT3 pathway inhibition [196]. Currently, FRA has been used as TCM preparations against malignancies in clinic.

### Phenylpropanoids

#### Coumarins

Notopterol (NOT), a furanocoumarin and primary bioactive component of *Hansenia weberbaueriana* (Fedde ex H.Wolff) Pimenov & Kljuykov, established efficacy in arthritis management. A research demonstrated that NOT directly bound to JAK2 and JAK3, inhibiting JAK-STAT signaling pathway activation [197]. And NOT also provided chondroprotective effects against inflammation through JAK2-STAT3 pathway suppression [198].

#### Lignans

Koreanaside A (KA), a lignan isolated from the flowers of *Forsythia koreana* (Rehder) Nakai, inhibited LPS-induced pro-inflammatory mediators in activated macrophages through JAK1/2 inactivation and subsequent STAT1/3 signaling pathway suppression [199]. KA also has a significant improvement effect on pathological manifestations of colitis, such as colon shortening, and spleen enlargement, positioning it as a potential therapeutic strategy for colitis.

### Steroids

Physalin A, a bioactive withanolide from *Alkekengi officinarum* var. franchetii (Mast.) R.J.Wang, was reported to exert anti-tumor activity in NSCLC and HCC [200, 201]. Physalin A modulated the tyrosine phosphorylation of JAK2 and JAK3 in a dose-dependent manner and abrogated the nuclear translocation and transcriptional activity of STAT3, showing anticancer activity in NSCLC [200]. But PI3K-AKT signaling pathway was considered to related to physalin A-induced apoptosis and autophagy in HCC [201].

### Polyethers

Salinomycin, a polyether antibiotic produced by *Streptomyces albus* via tank fermentation, overcomes tumor multidrug resistance by selectively targeting the cancer stem cells, positioning it as a novel chemotherapeutic agent [202]. Although the underlying mechanisms of the Salinomycin’s anticancer effects remained incompletely characterized, some evidence indicated that salinomycin decreased IFN-γ-induced IDO1 expression in human breast cancer cells through inhibiting the JAK-STAT pathway [203].

### Alkaloids

Matrine (MT), a quinolizidine alkaloid isolated from *Sophora flavescens* Aiton, exhibits broad pharmacological activities, such as anti-inflammatory, antitumor, anti-arrhythmic, antifibrotic, and cardioprotective effects [204–206]. MT suppressed the proliferation in human cholangiocarcinoma cells via inhibition of JAK2-STAT3 signaling pathway [207]. Oxymatrine (OMT), the oxide derivative of MT, inhibited tumor growth in a lung cancer xenograft model by blocking JAK1, JAK2 and Src kinase activation upstream of STAT5, thereby suppressing STAT5 phosphorylation [208].

## Conclusions and prospects

The objective of this review was to collect comprehensive information about potential JAK inhibitors from natural products. Hot compounds for research got more article length in this paper. For compounds with low expectation, we briefly state the status of the research. Through this review, we hope to provide natural products which can been screened as a therapeutic strategy for researchers and find some patterns that others can refer to.

In JAK-STAT signaling pathways, JAK1-STAT3 and JAK2-STAT3 got priority in molecular mechanisms research of potential JAK inhibitors. This can be attributed to extensive involvement of JAK1, JAK2, STAT3 in inflammatory response, immune response, tumorigenesis. In addition, JAK1-STAT5, JAK2-STAT1, JAK3, TYK2 are also directions in which we can promisingly make more progress.

In this review, JAK1, JAK2 and multi-target JAK inhibitors take up most of the article but JAK3 and TYK2 inhibitors take up few. We classify the compounds of the former according to their chemical structures, finding most belongs to terpenes and polyphenols. This suggests that these categories are easier to find biologically active ingredients and they deserve more attention. For plant extracts, although we didn’t find any rules that we can utilize in the classification, but most herbs are common medicine of TCM. This echoes what we have said above. These traditional Chinese herbs are a necessary treasure house for us to discover and invent new drugs.

According to our investigation, among autoimmune disorders, research hotspots focused on RA, colitis, AD. Among cancers, NSCLC, HCC and colon cancer got more attention.

Currently, the clinical translation of these natural JAK inhibitors still faces numerous challenges, with the developability issues of small molecule compounds remaining unresolved. Due to limited reported data on ADME properties of natural JAK inhibitors, we utilized a specialized platform (https://drugflow.com/) for predictive analysis (Supplementary Table 1). The predictions revealed that six compounds, including Ouabain and Crocin, exhibited excessive molecular weight, while nearly half showed LogP values outside the desirable range. Ultimately, 19 compounds such as Ouabain and Lycopene were excluded based on Lipinski’s Rule of Five. Additionally, 26 compounds including Lycopene and Ellagic Acid may suffer from low oral bioavailability, half of the compounds (e.g., Calcaratarin D and Cycloastragenol) might have T1/2 < 3 h, and most compounds potentially pose skin sensitization concerns. These results indicate significant challenges in transforming existing natural products into clinical therapeutics. However, we also identified several promising compounds with development potential, such as Igalan, Sanshool, Spilanthol, Geraniol, Fraxinellone, Matrine, and Psoralen, which demonstrate favorable drug-like properties and inhibitory activity, representing the most valuable candidates for clinical translation. Similarly, certain compounds, after proper modification, also hold significant application potential.

Throughout this review, we have known that the mechanism of action of many compounds remains to be further clarified and some remains controversial in effects on JAK. These leave the problems we need solve urgently. For drugs with high efficacy and safety, pushing them to the clinic is what we need to do in the medium and long term. We hope that this review will contribute to the discovery and development of new JAK inhibitors.

## Supplementary Information

Additional file 1.

## Data Availability

Not applicable.

## Abbreviations

AD: Atopic dermatitis
AMF: Amentoflavone
BA: Betulinic acid
BI: Baicalin
CalD: Calcaratarin D
CAG: Cycloastragenol
CGA: Chlorogenic acid
CN: D-carvone
CRC: Colorectal adenocarcinoma
DL: Darutigenol
Evo: Evodiamine
EGCG: Epigallocatechin gallate homoharringtonine
FRA: Fraxinellone
FT: Formononetin
GEN: Geniposide
HCC: Hepatocellular carcinoma
HDFs: Human dermal fibroblasts
HHT: Homoharringtonine
HMA: 2-Hydroxy-3-methylanthraquinone
ICA: Icariin
IBC: Isobavachalcone
JAK: Janus kinase
KA: Koreanaside A
LUT: Luteolin
MT: Matrine
NC: Nitidine chloride
NOT: Notopterol
NSCLC: Non-small cell lung cancer
PHI: Phillygenin
P-13: 2-Desoxy-4β-propylcarbamate-pulchellin
ROS: Reactive oxygen species
RA: Rheumatoid arthritis
RCC: Renal cell carcinoma
STAT: Signal transducer and activator of transcription
TCM: Traditional Chinese medicine
TQ: Thymoquinone
TPL: Triptolide
TSN: Toosendanin
WWOX: WW domain-containing oxidoreductase
5-DN: 5-Demethylnobiletin
